# Traditional Chinese medicine formula Xiaoqinglong decoction for cough caused by COVID-19: A protocol for a systematic review and meta-analysis

**DOI:** 10.1097/MD.0000000000023261

**Published:** 2020-11-25

**Authors:** Xiaodan Ren, Yu Shi, Guizhong Li

**Affiliations:** a The Second Affiliated Hospital of Army Medical University; b Beibei Traditional Chinese Medical Hospital, Chongqing, China.

**Keywords:** cough, COVID-19, systematic review, traditional Chinese medicine

## Abstract

**Background:**

Assessing the effectiveness and safety of Traditional Chinese medicine formula Xiaoqinglong decoction for cough caused by COVID-19 is the main purpose of this systematic review protocol.

**Methods:**

The following electronic databases will be searched from their respective inception dates to October 1, 2020: PubMed, MEDLINE, the Cochrane Library, Embase, WorldSciNet, Ovid, the Allied and Complementary Medicine Database, the Web of Science, Chinese Biomedical Literature Database, China National Knowledge Infrastructure, the Chongqing VIP Chinese Science and Technology Periodical Database (VIP), the Wanfang Database, and the China Biology Medicine Disc. All published randomized controlled trials in English or Chinese related to Traditional Chinese medicine formula Xiaoqinglong decoction for cough caused by COVID-19 will be included. The primary outcome is the time and rate of appearance of coughing. The secondary outcomes are the length of hospital stay. Two reviewers will conduct the study selection, data extraction, and assessment independently. The assessment of risk of bias and data synthesis will be conducted with RevMan V.5.2.

**Results:**

The results will provide a high-quality synthesis of current evidence for researchers in this subject area.

**Conclusion:**

The conclusion of our study will provide an evidence to judge whether traditional Chinese medicine formula Xiaoqinglong decoction is an effective intervention for patients with cough caused by COVID-19.

**Ethics and dissemination:**

Formal ethical approval is not necessary as the data cannot be individualized. The results of this protocol will be disseminated in a peer-reviewed journal or presented at relevant conferences.

**PROSPERO registration number:**

CRD42020202079.

## 1 Introduction

An epidemic of acute respiratory syndrome in humans, named as “Coronavirus Disease 2019” (COVID-19), appeared in Wuhan, China in December 2019.^[1]^ There were many reports related to a live-animal and seafood market, supporting that the pathogens were transferred from animals to humans, rapidly evolving into transmission from human to human and the pathogen was classified as 2019 Novel Corona Virus (2019-nCoV).^[2]^ COVID-19 is spreading very fast and as of February 19, 2020, this rose to a total of 74,280 confirmed cases in China and 924 confirmed cases in 24 countries outside China, and a total of 2009 deaths globally.^[3]^ This new kind of disease could cause symptoms including fever, cough, and myalgia or fatigue.^[4]^ In diagnosis, real-time reverse transcription polymerase chain reaction of viral nucleic acid is regarded as the reference standard.^[5]^ For the treatment, there is no any specific effective antiviral treatment and optimized supportive care remains the mainstay of therapy.^[6]^ Respiratory system symptom, especially cough, is the primary one. As a result of the fact that there is no specific therapy for this new virus, dealing with the patient symptomatically becomes the only choice for the clinicians.^[7]^

Traditional Chinese medicine, originating from China thousand years ago, has been playing a significant role in the treatment of COVID-19.^[8]^ Xiaoqinglong decoction (XQL) is a famous prescription used to treat diseases of respiratory system including cough, asthma, and COPD.^[9]^ It consists of 8 herbal materials: ephedra herb, Radix Paeoniae Alba, Asarum dirboldii Mig, Rhizoma Zingiberis, honey-fried licorice root, cassia twig, Schisandra chinensis, and Phinellia ternata. Clinical trials and animal models have proven the effectiveness of XQL on COPD and cough of other pathogens.

## 2 Methods and analysis

### 2.1 Study registration

This systematic review protocol was registered with PROSPERO 2020 (registration number: CRD42020202079). And the protocol report is in the base of the Preferred Reporting Items for Systematic Reviews and Meta-Analyses Protocols (PRISMA-P) declaration guidelines.^[10]^ The review will be performed in line with the PRISMA-P declaration guidelines.^[11]^

### 2.2 Inclusion criteria for study selection

#### 2.2.1 Type of study

All randomized controlled trials (RCTs) about traditional Chinese medicine formula Xiaoqinglong decoction for cough caused by COVID-19 which were reported in English and Chinese will be included. Trials with 2-arm or 3-arm parallel design will be also included. Non-RCTs, quasi-RCTs, case series, reviews, animal studies, and any study with a sample size of less than 10 participants will be excluded.

#### 2.2.2 Type of participant

Participants who were 18 years or older with cough caused by COVID-19 will be included in spite of the gender, race, education, or economic status.

#### 2.2.3 Type of intervention

Experimental interventions include Traditional Chinese medicine therapy. Control interventions would be western medicine therapy.

#### 2.2.4 Type of outcome measure

The primary outcome will be the time and rate of appearance of coughing. The secondary outcomes will be the length of hospital stay.

### 2.3 Search methods for identification of studies

#### 2.3.1 Electronic data sources

The following electronic databases will be searched from their respective inception dates to October 1, 2020: PubMed, MEDLINE, the Cochrane Library, Embase, WorldSciNet, Ovid, the Allied and Complementary Medicine Database, the Web of Science, Chinese Biomedical Literature Database, China National Knowledge Infrastructure, the Chongqing VIP Chinese Science and Technology Periodical Database (VIP), the Wanfang Database, and the China Biology Medicine Disc. All published randomized controlled trials in English or Chinese related to Traditional Chinese medicine formula Xiaoqinglong decoction for cough caused by COVID-19 will be included.

### 2.4 Searching other resources

The reference lists of potentially missing eligible studies will be scanned and the relevant conference proceedings will be scanned as well.

### 2.5 Search strategy

The search strategy for PubMed is shown in Table 1. The following search keywords will be used: Traditional Chinese medicine (e.g., “Chinese Drugs, Plant” or “Chinese Herbal Drugs” or “Herbal Drugs, Chinese” or “Plant Extracts, Chinese” or “Chinese Plant Extracts” or “Extracts, Chinese Plant”); Xiaoqinglong decoction; cough; COVID-19 (e.g., “2019-nCoV” or “Wuhan coronavirus” or “SARS-CoV-2” or “2019 novel coronavirus” or “COVID-19 virus” or “coronavirus disease 2019 virus” or “COVID19 virus” or “Wuhan seafood market pneumonia virus”); randomized controlled trial (e.g., “randomized controlled trial” or “controlled clinical trial” or “random allocation” or “randomized” or “randomly” or “double-blind method” or “single-blind method” or “clinical trial”. The equivalent search keywords will be used in the Chinese databases.

**Table 1:** Search strategy for the PubMed database.

Number	Search items
1	COVID-19. Mesh.
2	COVID-19.ti, ab
3	Wuhan coronavirus.ti, ab
4	SARS-CoV-2.ti, ab
5	2019 novel coronavirus.ti, ab
6	COVID-19 virus.ti, ab
7	Coronavirus disease 2019 virus.ti, ab
8	COVID19 virus.ti, ab
9	Wuhan seafood market pneumonia virus.ti, ab
10	1 or 2–10
11	cough. Mesh.
12	cough. ti, ab
13	11 or 12
14	Xiaoqinglong decoction. Mesh.
15	Xiaoqinglong decoction. Ti,ab
16	14 or 15
17	Traditional Chinese medicine. Mesh.
18	Chinese Drugs, Plant. ti, ab
19	Chinese Herbal Drugs. ti, ab
20	Herbal Drugs, Chinese. ti, ab
21	Plant Extracts, Chinese. ti, ab
22	Chinese Plant Extracts. ti, ab
23	17 or 18–22
24	Randomized controlled trial. pt
25	Controlled clinical trial. pt
26	Randomized controlled trials. Mesh.
27	Random allocation. Mesh.
28	Randomized. ti, ab
29	Randomly. ti, ab
30	Double-blind method. Mesh
31	Single-blind method. Mesh
32	Clinical trial. pt
33	24 or 25–32
34	10 and 13 and 16 and 23 and 33

### 2.6 Data collection and analysis

#### 2.6.1 Selection of studies

**Figure 1:**
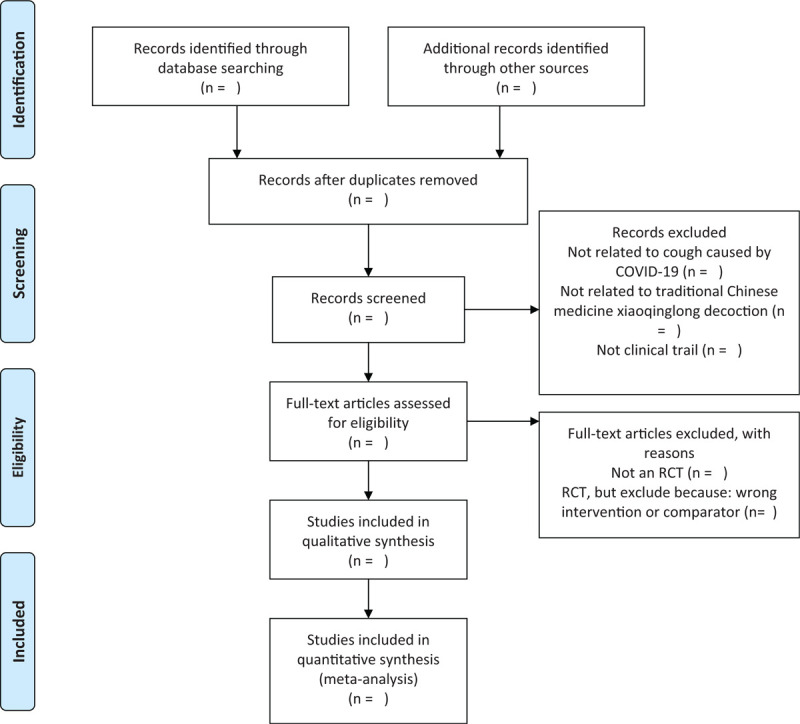
The PRISMA-P flow chart of the study selection process.

The titles and abstracts of all searched studies will be reviewed and screened independently by 2 reviewers, aiming at identifying eligible trials and eliminating duplicated or irrelevant studies in line with the criteria; the full text of all possibly eligible studies will be obtained if necessary. A discussion with the third reviewer is planned to solve the disagreements. A PRISMA-P flow diagram will be used to show the study selection process (Fig. 1).

#### 2.6.2 Data extraction and management

The following data will be extracted from the selected studies by 2 independent reviewers using a standard data extraction sheet: year of publication, country, general information, participant characteristics, inclusion and exclusion criteria, sample size, randomization, blinding methods, methods, type of acupuncture interventions, control, outcome measures, results, adverse reactions, conflicts of interest, ethical approval, and other information. The authors will be contacted for further information if the reported data is insufficient and the third reviewer will be set to solve the disagreements.

#### 2.6.3 Assessment of risk of bias and reporting of study quality

Two independent reviewers will access the quality of included literature and complete the Standards for Reporting Interventions in Clinical Trials of Acupuncture checklist with the Cochrane collaboration risk-of-bias assessment method.^[12]^

#### 2.6.4 Measures of treatment effect

Dichotomous data will be presented as risk ratio and 95% confidence intervals (CI), while continuous outcomes will be shown as standard mean difference 95% CI.

#### 2.6.5 Unit of analysis issues

The individual participant will the analytical unit.

#### 2.6.6 Management of missing data

The cause of the missing data will be determined to solve the problem. And if this is not working, the authors will be contacted for the missing part. This will be documented and the available data will be extracted and analyzed if the missing data cannot be obtained.

#### 2.6.7 Assessment of heterogeneity

I^2^ test will be used to quantify inconsistency and standard χ^2^ test will be used to detect statistical heterogeneity. Studies will be considered to have homogeneity if the *P* value exceeds.1 and the I^2^ value is less than 50%, and the fixed-effects model will be used. While studies will be considered to have significant statistic heterogeneity if the *P* value is less than .1 or the I^2^ value exceeds 50%, and subgroup analysis will be used to explore the possible cause. And the random-effects model will be applied if the heterogeneity is still important.

#### 2.6.8 Assessment of reporting biases

Funnel plots will be used to assess the reporting biases if more than 10 studies are included.

### 2.7 Data synthesis

RevMan V.53 will be used for data synthesis. The level of statistical heterogeneity will determine how the data will be synthesized and analyzed. The random-effects model will be used if the I^2^ value is no less than 50%. The fixed-effects model will be used if the heterogeneity tests show little statistical heterogeneity. If there is meaningful heterogeneity that cannot be explained by any assessment, meta-analysis will not be performed. If necessary, each subgroup will be carefully considered for subgroup analysis.

### 2.8 Subgroup analysis

Subgroup analysis will be conducted if the data are sufficient, according to the factors different outcomes and different control interventions.

### 2.9 Sensitivity analysis

Sensitivity analysis will be conducted to test the robustness of the review conclusions if possible. The impacts of sample size, study design, methodological quality, and missing data will be evaluated.

### 2.10 Grading the quality of evidence

The Grade of Recommendations Assessment, Development and Evaluation will be the tool to evaluate the quality of the evidence.^[13]^ Limitation of study design, inconsistency of results, indirectness, imprecision, and publication bias will be assessed. The assessments will be divided into four levels: Very low, low, moderate, or high.

### 2.11 Ethics and dissemination

This protocol will not evaluate individual patient information or affect patient rights and therefore does not require ethical approval. Results from this review will be disseminated through peer-reviewed journals and conference reports.

## 3 Discussion

This systematic review will assess the effectiveness and safety of Traditional Chinese medicine formula Xiaoqinglong decoction for cough caused by COVID-19. There are four sections in the review: identification, study inclusion, data extraction, and data synthesis. This review will help the doctors to choose Traditional Chinese medicine formula Xiaoqinglong decoction as an alternative treatment for cough caused by COVID-19, and offer the patients more options to relieve their symptoms.

## Author contributions

XR and YS mainly contributed to this manuscript and joint first authors. GL obtained funding. XR drafted the protocol. YS make the search strategy. XR will obtain copies of the studies and screen the studies to be included. Data extraction from the studies will be done by GL. YS will put the data into RevMan. Analyses will be conducted by XR. XR will draft the final review and GL will update the review. GL will act as an arbiter in the study selection stage. All authors have read and approved the final manuscript.

**Data curation:** Guizhong Li.

**Formal analysis:** Guizhong Li.

**Funding acquisition:** Guizhong Li.

**Methodology:** Yu Shi.

**Software:** Yu Shi.

**Writing – original draft:** Xiaodan Ren.
